# A case of Miller Fisher syndrome with delayed onset peripheral facial nerve palsy after COVID-19 vaccination: a case report

**DOI:** 10.1186/s12883-022-02838-4

**Published:** 2022-08-22

**Authors:** Kentaro Nanatsue, Makoto Takahashi, Sakiko Itaya, Keisuke Abe, Akira Inaba

**Affiliations:** Department of Neurology, Kanto Central Hospital, 6-25-1 Kami-Yoga, Setagaya-ku, Tokyo, 158-8531 Japan

**Keywords:** Fisher syndrome, Miller Fisher syndrome, Anti-GQ1b antibody, COVID-19, Post-vaccination, Moderna, IVIg, Bell’s palsy, Case report

## Abstract

**Background:**

To prevent the spread of the novel coronavirus disease 2019 (COVID-19) infection, various vaccines have been developed and used in a large number of people worldwide. One of the most commonly used vaccines is the mRNA vaccine developed by Moderna. Although several studies have shown this vaccine to be safe, the full extent of its side effects has not yet been known. Miller-Fisher syndrome (MFS) is a rare condition that manifests ophthalmoplegia, ataxia, and loss of tendon reflexes. It is a subtype of Guillain–Barré syndrome and an immune-mediated disease related to serum IgG anti-GQ1b antibodies. Several vaccines including those for COVID-19 have been reported to induce MFS. However, there have been no reports of MFS following Moderna COVID-19 vaccine administration.

**Case presentation:**

A 70-year-old man was referred to our hospital due to diplopia that manifested 1 week after receiving the second Moderna vaccine dose. The patient presented with restricted abduction of both eyes, mild ataxia, and loss of tendon reflexes. He was diagnosed with MFS based on his neurological findings and detection of serum anti-GQ1b antibodies. The patient was administered intravenous immunoglobulin, and his symptoms gradually improved. Five days after admission, the patient showed peripheral facial paralysis on the right side. This symptom was suggested to be a delayed onset of peripheral facial nerve palsy following MFS that gradually improved by administration of steroids and antiviral drugs.

**Conclusion:**

There have been no previous reports of MFS after Moderna COVID-19 vaccination. This case may provide new information about the possible neurological side effects of COVID-19 vaccines.

## Background

Four types of COVID-19 vaccines have been approved by the pharmaceutical authorities in Japan: the Pfizer-BioNTech, Moderna, Oxford/AstraZeneca, and Novavax/Takeda. The first two, including the one developed by Moderna, were the first mRNA vaccines in history. The adverse reactions of mRNA vaccines comprise soreness at the vaccination site, fatigue, headache, and muscle pain, which are thought to be side effects of immune activation [1]. However, these side effects have not been yet fully understood.

Miller Fisher syndrome (MFS) is an immune-mediated disease characterised by acute external ophthalmoplegia, ataxia, and loss of tendon reflexes [2]. MFS is a known subtype of Guillain-Barré syndrome (GBS). Serum anti-GQ1b antibodies, a type of anti-ganglioside antibody, have been reported to be positive in 80–90% of patients with MFS [3]. MFS has been reported to be triggered by factors that affect immunity, such as prior infection or lymphoma. Vaccination including those used for COVID-19 is also considered to be one of these factors. However, the Moderna COVID-19 vaccine is currently not known to cause MFS.

Herein, we report a case of a 72-year-old Japanese man who presented with MFS and delayed-onset peripheral facial nerve palsy after administration of the Moderna COVID-19 vaccine.

## Case presentation

A 72-year-old Japanese man was admitted to our department with diplopia that he experienced the night before. The next morning, since his diplopia did not improve, the patient visited our hospital. The patient had no history of allergies. However, he had a history of surgery for aortic regurgitation and was prescribed warfarin. He also had hypertension, hyperlipidaemia, and hyperuricemia during medical treatment. Although his father had von Recklinghausen’s disease (VRD) type I, the patient showed no obvious symptoms suggestive of VRD.

He received his first and second doses of the Moderna vaccine four and 1 weeks prior to his visit, respectively. No obvious symptoms were observed between the first and second vaccinations. The day after the second dose of vaccination, he developed a fever of 38 °C, sore throat, and runny nose, which improved spontaneously within a few days after acetaminophen administration. There were no other infectious symptoms, including gastrointestinal ones.

**Fig. 1:**
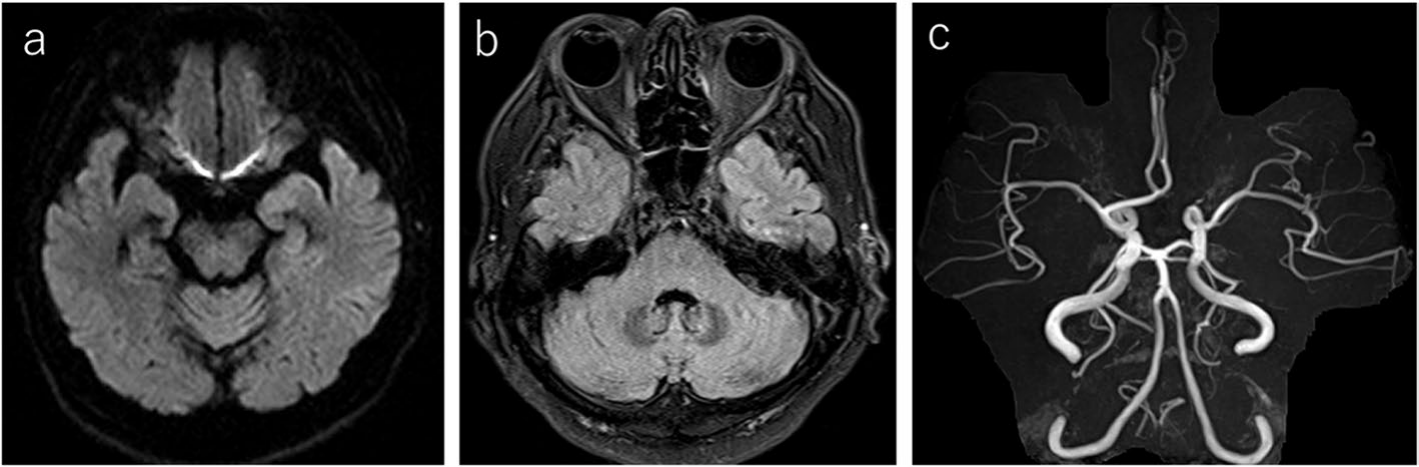
**a** Diffusion-weighted magnetic resonance images, **b** Fluid-attenuated inversion-recovery images, and **c** Magnetic resonance angiography shows no abnormalities

Neurological examination revealed mild ocular motor restriction in all directions with bilateral adduction, ataxia of the limbs and trunk, and loss of tendon reflexes in all extremities. No muscle weakness or sensory disturbances were noted. Blood test results, including glucose, thyroid function, and anti-acetylcholine receptor antibodies, were normal, except for prolongation of the prothrombin time by warfarin. Polymerase chain reaction (PCR) test for COVID-19 was negative. Head magnetic resonance imaging and magnetic resonance angiography showed no obvious abnormal findings, including cerebral infarction or aneurysm (Fig. 1a, b, c). On the day after admission, the patient’s eyes were fixed at the midline. His ataxia worsened, causing him difficultly in walking unaided. MFS was suspected based on clinical symptoms; previous examinations; and differential diagnosis to exclude diseases including brainstem vasculopathy, cerebral aneurysm, Wernicke syndrome, multiple sclerosis, neuromyelitis optica, and myasthenia gravis. Therefore, intravenous immunoglobulin (IVIg) therapy was performed. On the third day, weakness in proximal muscles such as the deltoid and iliopsoas appeared. Nerve conduction studies revealed F-wave abnormalities. His ataxia and muscle weakness peaked on the fifth day and gradually improved thereafter. On the sixth day, serum anti-GQ1b antibodies were detected, and the patient was diagnosed with MFS. Sputum and stool cultures did not detect any bacteria associated with MFS.

On the seventh day, he developed right-sided peripheral facial nerve palsy. Head magnetic resonance imaging revealed no abnormalities. Steroids, valacyclovir, and mecobalamin were administered, considering the possibility of Bell’s palsy.

These treatments gradually improved all symptoms. His muscle weakness and ataxia almost disappeared, and he was discharged home on the 22nd day. One month after hospital discharge, the other symptoms, including diplopia and facial nerve palsy, also disappeared.

## Discussion and conclusions

Our case highlights three considerations: the validity of the patient’s diagnosis as MFS; relationship between MFS and peripheral facial nerve palsy; and link between COVID-19 vaccination, MFS, and peripheral facial nerve palsy.

MFS is diagnosed based on clinical findings of acute external ophthalmoplegia, ataxia, and loss of tendon reflexes with other diseases exclusion [2]. Serological positivity for anti-GQ1b antibodies [3], which is seen in about 90% of MFS cases, is an important adjunct in diagnosis. In our case, the patient showed all three symptoms previously mentioned and serological detection of anti-GQ1b antibodies, which led to a diagnosis of MFS. Although the patient showed muscle weakness during the disease course, this manifestation is known to occur in about 10% of MFS cases [4] and seems consistent with MFS. MFS has a good natural history of recovery and does not necessarily require immunotherapy. However, in our case, since the patient was an older adult and showed rapid progression with muscle weakness, IVIg therapy was administered and led to rapid improvement.

In this case, right peripheral facial nerve palsy was observed on the seventh day of hospitalisation, after MFS symptoms improved. Although cerebral infarction, cerebellar bridge angle tumour associated with VRD type II, and Ramsay–Hunt syndrome were considered as possible differential causes, they were ruled out based on the physical and laboratory findings. Thirty-two percent of patients with MFS are known to have subsequent facial nerve palsy [5], and some of them (6–16%) have recurrent facial nerve palsy that develops during the period of symptom improvement [6–8]. In this case, the possibility of idiopathic facial nerve palsy (Bell’s palsy) could not be ruled out. However, based on the course of the case, delayed peripheral facial nerve palsy, associated with MFS, was considered the most likely cause.

Lastly, we evaluated the association between vaccination for COVID-19, MFS, and peripheral facial nerve palsy. Prior infections are found in about 80% of MFS cases and assumed to trigger immune abnormalities [6]. *Haemophilus influenzae*, which causes upper respiratory tract infection, and *Campylobacter jejuni*, are known to be major causative agents of MFS. These bacteria are known to have a GQ1b-like structures in their outer membrane [9, 10]. MFS after COVID-19 infection has also been reported [11]. In a report of 12 cases of MFS after COVID-19 infection, six cases had onset within 7 days after infection and only five cases were positive for anti-ganglioside antibody. Although ten patients showed mild to complete improvement, one case was severe enough to require intubation and another had sudden death due to arrhythmia. In our patient, the PCR test for COVID-19 was negative on admission and there were no common cold symptoms before the disease onset. Therefore, we concluded that the patient did not have COVID-19-associated MFS. Triggers for MFS development other than infections include TNF-α inhibitors use, Burkitt’s lymphoma, and Hodgkin’s lymphoma, which are also thought to affect immunity [12]. Vaccination, which primes the natural immune system against possible attacks of certain viruses and bacteria, may also trigger MFS. Cases of MFS after influenza and pneumococcal vaccinations have been reported [13, 14]. Four cases of MFS caused by COVID-19 vaccination except for our case also have been reported (Table 1) [15–18]. All four cases were reported after receiving the Pfizer vaccine. To date, our case is the first case of MFS following Moderna vaccine administration. In all five cases including our case, the time from vaccination to disease onset was at least 7 days. Additionally, GQ1b antibodies were detected in three of the four cases tested. All five patients were treated with IVIg therapy, and four patients achieved remission (The course of one case was not reported). Compared with MFS caused by COVID-19 infection, COVID-19 vaccine-associated MFS is more likely to have a later onset (> 7 days), and may have a higher rate of anti-ganglioside antibody positivity. Although most patients in both cases showed good improvement, one case of MFS after COVID-19 infection was severe and another case was fatal, suggesting that MFS after COVID-19 infection is rare but can be severe.

**Table 1:** Cases with Miller Fisher Syndrome after COVID-19 vaccination

Age	Sex	Nationality	Type of Vaccine	Number of vaccinations	Time from vaccine to onset (days)	Past history	Neurological findings	CSF cells(/ul) and Protein (mg/dl)	Anti-ganglioside antibodies	NCS	Treatment	Outcome	Reference
30	M	Japan	Pfizer BNT162b2	2	7	none	bilateral gaze palsy, areflexia, ataxia	1 / 30.8	GQ1b, GT1a	normal	IVIg	recovered	[15]
71	M	Japan	Pfizer BNT162b2	2	16	DM	ptosis, loss of light reflex, oculomoter nerve palsy, ataxia	1 / 67	negative	normal	IVIg	recovered	[16]
24	F	N.R.	Pfizer BNT162b2	1	18	none	impaired abduction and elevation of both eyes	2 / 296 (albmin) (mg/L)	GQ1b	normal	IVIg	recovered	[17]
37	M	Syrian	Pfizer BNT162b2	1	9	none	dysphagia, dysarthria	N.R.	N.R.	N.R.	IVIg	N.R.	[18]
72	M	Japan	COVID-19 Moderna	2	7	HT, HL, HUA, AR	diplopia, ataxia	N.D.	GQ1b	F-wave abnormalities	IVIg	recovered	our case

*M* Male, *F* Female, *N.R.* Not reported, *DM* Diabetes mellitus, *HT* Hypertension, *HL* Hyperlipidemia, *HU* Hyperuricemia, *AR* Aortic regurgitation, *CSF* Cerebrospinal fluid, *N.D.* Not determined, *NCS* Nerve conduction study, *IVIg* Intravenous immunogloblin therapy

In our case, symptoms of possible adverse reactions to the Moderna COVID-19 vaccine appeared 1 day after the second dose of vaccine, and MFS developed 1 week later. Since the patient had sore throat and runny nose in addition to fever, we could not completely deny the possibility that the upper respiratory infection occurred at the same time of the vaccination. However, sore throat and runny nose have been reported at a frequency of 6.02 and 2.78%, respectively, after administration of the Moderna vaccine [19]. In addition, no bacteria associated with MFS were detected in the patient’s sputum culture. For these reasons, it is possible that the vaccination might have triggered his MFS by immunological activation. We instructed the patient to avoid receiving the third vaccine dose as much as possible, depending on the surrounding infection status. In addition if the patient had to receive this third dose, he should have avoided the Moderna vaccine.

It is also known that immune-mediated or viral reactivation can cause facial paralysis after vaccination [20]. There is also a report of Bell’s palsy after receiving the Moderna COVID-19 vaccine [21]. However, the frequency is very rare, at 7 out of 35,654 according to a Phase III trial [22]. Due to the different timing of MFS onset and facial nerve palsy, and the fact that delayed peripheral nerve palsy after MFS is known, peripheral nerve palsy seemed to be more related to MFS than to the vaccine.

To our knowledge, this is the first case of MFS followed by peripheral facial nerve palsy after receiving the Moderna COVID-19 vaccine. Based on the medical history and laboratory results, it was considered that the COVID-19 vaccine may have caused that MFS. It seems necessary to pay attention to the appearance of neurological findings related to MFS, such as diplopia and light-headedness, after receiving COVID-19 vaccination.

## Data Availability

Not applicable.

## Abbreviations

COVID-19: Coronavirus disease
MFS: Miller Fisher syndrome
GBS: Guillain–Barré syndrome
PCR: Polymerase chain reaction
VRD: Von Recklinghausen’s disease
